# Editorial: Novel phenotyping and risk stratification strategies for heart failure

**DOI:** 10.3389/fcvm.2022.1115991

**Published:** 2023-01-05

**Authors:** Jeffrey Shi Kai Chan, Ana Ciobanu, Ying Liu, Aggeliki Gkouziouta, Tong Liu

**Affiliations:** ^1^Heart Failure and Structural Heart Disease Research Unit, Cardiovascular Analytics Group, Hong Kong, Hong Kong SAR, China; ^2^Faculty of Medicine, Carol Davila University of Medicine and Pharmacy, Bucharest, Romania; ^3^Department of Internal Medicine and Cardiology, Theodor Burghele Clinical Hospital, Bucharest, Romania; ^4^Heart Failure and Structural Cardiology Division, The First Affiliated Hospital of Dalian Medical University, Dalian, Liaoning, China; ^5^Onassis Cardiac Surgery Center, Athens, Greece; ^6^Tianjin Key Laboratory of Ionic-Molecular Function of Cardiovascular Disease, Department of Cardiology, Tianjin Institute of Cardiology, Second Hospital of Tianjin Medical University, Tianjin, China

**Keywords:** heart failure, phenotype, risk stratification, epidemiology, pathophysiology

Since its designation as an emerging epidemic in 1997, heart failure (HF) has remained a major public health problem (1). With an estimated 64.3 million people living with HF worldwide, it is a common cause of hospitalizations and contributes significantly to healthcare costs, morbidity, and mortality (2). Although recent decades have seen massive leaps in the understanding and management of HF, much remains to be explored and the frontiers of HF research continue to be pushed, as is evident from the 15 excellent articles presented in this Research Topic.

Pathophysiological understanding is critically important in all medical conditions, and HF is no exception. Here, Meng et al. presented a prospective cohort of 84 consecutive patients with acute decompensation of HF who, compared to 83 patients without HF, had higher CD4^+^/CD8^+^ expression of T cell immunoglobulin and mucin domain-containing protein 3 (Tim-3), a unique inhibitory co-receptor expressed on the surface of immune cells and mediates immune tolerance; Tim-3 expression was also independently associated with major adverse cardiac and cerebrovascular events. These highlighted the importance of inflammation in acute decompensation of HF. Meanwhile, Li N. et al. provided a comprehensive review of the role of RNA binding motif protein 20 (RBM20) and other splicing factors in titin isoform ratio modification, myocardial stiffness, and thus pathophysiology of HF with preserved ejection fraction (HFpEF). Aside from pathophysiological insights, the authors suggested that RBM20 may be a therapeutic target for mitigating myocardial stiffness in HFpEF, which, given the relative paucity of efficacious treatments for HFpEF (3), may be an exciting prospect warranting further exploration.

These recent advances in our pathophysiological understanding of HF also implied new opportunities to better characterize HF, with recent years having seen many novel risk factors and diagnostic tools being identified (4–7). Knowing that the diagnosis and workup of HFpEF is particularly challenging (8), Lau et al. provided a concise but informative summary of the role of cardiac magnetic resonance imaging in the assessment of HFpEF, which should be useful for both clinicians and researchers alike. However, the term “HFpEF” also points to the issue of phenotyping: even though left ventricular ejection fraction has been the most common way of classifying HF phenotypes, it may be a relatively insensitive marker of myocardial function, may not adequately reflect myocardial dysfunction, and has considerable temporal variability and operator dependency, amongst other limitations (9). Thus, alternative means of classifying and phenotyping HF have been extensively explored and remain an active area of research. Here, Sun et al. summarized studies that used clustering analysis for discovering new HF phenotypes. They found that patients from Africa, South America, and South and West Asia were under-represented, and that studies with a large number of clustering variables tended to have small sample sizes which may be statistically detrimental. There was also an under-exploration of functional outcomes as endpoints, and a lack of exploration of genomic and proteomic data, which may represent new opportunities for further studies.

Amongst those diagnosed with HF, prognostication remains to be of much clinical interest. In prospective multicentre cohort study of 4,305 Chinese patients with HF, Ge et al. found an inverse association between lean body mass and mortality, but none between fat mass and mortality. This relates to the contentious “obesity paradox”, a phenomenon where obese patients were observed to have lower mortality, contrary to common expectation (10). While some had raised the possibility of such “paradox” being a spurious association arising from collider bias (11), others have shown that collider bias only explains such “paradox” partially (12). In the case of HF, frailty/sarcopenia or cardiorespiratory fitness were possible confounders that could constitute collider bias. In this study by Ge et al., a higher lean body mass could be seen as a surrogate for the absence of sarcopenia, and collider bias was unlikely to have affected the findings. Overall, this study furthered our understanding of the interactions between body composition and HF outcomes.

Meanwhile, Zhao et al. showed, in a retrospective cohort study of 170 patients with myocarditis, that higher levels of N-terminal pro-hormone brain natriuretic peptide (NT-proBNP) were independently associated with higher risk of major adverse cardiovascular events (MACE), and that NT-proBNP was superior to left ventricular EF in predicting 30-day death or heart transplantation. In another biomarker study, Zong et al. studied 956 Chinese patients with HF and 485 without HF, and found that high levels of trimethyllysine (TML), a precursor to trimethylamine N-oxide (TMAO) which is a metabolic product of intestinal microorganisms, was independently associated with HF and positively correlated with levels of NT-proBNP. Furthermore, higher levels of TML was associated with the composite outcome of cardiovascular mortality and HF hospitalization amongst patients with HF. On a related note, Li X. et al. showed in a systematic review and meta-analysis of 10 observational studies with 13,425 patients that higher levels of TMAO were associated with MACE and mortality in HF; significant heterogeneity was observed for both outcomes, which was not unexpected given the observational nature of included studies and the varying definitions of elevated TMAO. Overall, these two latter studies gave insights into gut microbiota metabolites as novel prognosticators in HF, and had potential implications in gut-heart interactions that may contribute to the pathophysiology of HF. The mechanisms underlying the above observations remained to be elucidated, highlighting the importance of gut microbiota as a new frontier in HF research and a potential treatment target in HF.

While most treatments of HF are pharmacological, recent years have seen a number of devices emerging as promising therapeutic options. Here, Miyagi et al. reviewed novel device-based approaches to left atrial pressure relief, highlighting both the potential and limitations of this new frontier in personalized HFpEF management. This personalized approach to HF management was echoed by Guo et al., who explored associations between single-nucleotide polymorphisms of low-density lipoprotein receptor-related protein 6 (LRP6) and risks of sudden cardiac death and mortality, finding that the A allele of rs2302684 was associated with increased risks of these endpoints. Such finding has potential implications for personalized sudden cardiac death risk stratification and polygenic risk scores in HF. Combining genetic data with other markers, such as electrocardiographic and echocardiographic measurements (5, 13–15), may also improve predictive power.

Two studies also explored the effects of non-cardiovascular comorbidities and complications. In a retrospective, propensity score-matched cohort of 4,328 patients with HF without thyroid disease, Wang C. et al. found that a low FT3/FT4 ratio was associated with higher risks of all-cause and cardiovascular mortality. This adds to our understanding of the intricate interactions between thyroid and HF and suggests that clinicians may consider working up patients with HF and without overt thyroid diseases for subclinical thyroid dysfunction. On the other hand, Zhong et al. studied 100 patients with myocardial infarction in a case-control study, finding that low baseline hemoglobin was an independent risk factor for in-hospital post-myocardial infarction gastrointestinal bleeding, and that low hemoglobin and Kilip class IV were independent risk factors for in-hospital mortality in those who had such bleeding. These findings may aid clinicians in risk stratification of hospitalized patients with myocardial infarction.

Last but definitely not least, three studies delved into more specific populations with or at risk of HF for which evidence has been relatively scarce. In a retrospective cohort study of 306 Chinese patients with lung cancer, Ren et al. demonstrated that atrial cardiomyopathy was prevalent regardless of histological subtypes, and that atrial cardiomyopathy was associated with worse survival. With recent advancements in the understanding of cancer therapy-related cardiotoxicity (16–22), these are important findings that will facilitate risk stratification and management of patients with lung cancer. Grupper et al., on the other hand, studied 59 consecutive patients implanted with the HeartMate3 left ventricular assist device (LVAD) in a prospective cohort study, observing that a diastolic plateau, which is a sign observed during right heart catheterization and is typically associated with constrictive or restrictive pathologies, was associated with increased risks of adverse cardiovascular events. With right heart failure being the major cause of morbidity and mortality in patients receiving LVAD (23), these findings give insights into the haemodynamic effects of LVAD and may facilitate clinicians in the risk stratification of patients receiving LVAD. Meanwhile, Wang S. et al. described in great detail the presentation, phenotype, genetic mutations, investigation findings, and outcome of 29 Chinese patients with hereditary transthyretin amyloid cardiomyopathy. Hereditary transthyretin amyloid cardiomyopathy is likely underdiagnosed (24), and with the emergence of several novel, evidence-based treatments (25–27), this study was a timely contribution to our understanding of these patients.

To conclude, the 15 excellent articles in this Research Topic explored various aspects of HF research, with particular emphasis on phenotyping and risk stratification. We believe that these are valuable contribution to the literature that will better our understanding and management of HF in the years to come.

## Author contributions

All authors listed have made a substantial, direct, and intellectual contribution to the work and approved it for publication.
